# A Gruesome Controversy

**Published:** 1921-02-05

**Authors:** 


### A Gruesome Controversy.

An ex-soldier who is offering to "sell for transfer" any limb or other member of his body has provoked a curious controversy as to the legal rights possessed by a human being over his own body, either before or after death. This man's explanation of his amazing advertisement is that he is "down and out," and the sale of his limbs is his last resource. He has had some experience already, having suffered blood transfusion for four different persons, one of them a well-known peer. But the possibilities of blood transfusion as a means of livelihood are, as a sympathetic writer and the ex-soldier rather needlessly point out, distinctly limited. Learning that a prominent financier had lost an eye, he wrote offering one, but had no response. He says, quite seriously that "all orders for limbs will be executed in strict rotation."

According to the *Manchester Guardian*, a medical expert consulted on the point by an earnest inquirer, declared that there would be legal difficulties in the way. The legal point now being argued with much vivacity is in relation to the cognate theme of the disposal of one's corpse for anatomical purposes. A peer recently wrote to the papers pointing out that bodies were scarce, and that it is a pity that it is illegal for a man to give, devise, or bequeath his body for the purposes of science. But is not the peer in error? An authority of one of the London hospitals states that it is perfectly legal to leave your body in your will for anatomical research, but the will cannot be enforced unless a legacy is annexed to the direction. What can be done is to make the Public Trustee the executor and direct that your body shall be given to a hospital for dissection. It is asserted that people in want of money frequently go to the hospitals and offer to sell their bodies for £5, "the supposed market price." The hospital authorities never buy under these conditions. They get their subjects partly from inmates who die in hospital and partly from bequests. There are good reasons why it is in the public interest that transactions in the sale of a man's body, even by himself, should be under strict legal limitations.

